# Intensidade de Exercício durante o Teste de Caminhada de 6 Minutos em Pacientes com Doença Arterial Periférica

**DOI:** 10.36660/abc.20190053

**Published:** 2020-04-06

**Authors:** Breno Quintella Farah, Raphael Mendes Ritti-Dias, Polly Montgomery, Gabriel Grizzo Cucato, Andrew Gardner

**Affiliations:** 1 Universidade Federal Rural de PernambucoRecifePEBrasilUniversidade Federal Rural de Pernambuco, Recife, PE – Brasil; 2 Universidade Nove de JulhoPrograma de Pós-Graduação em Ciências da ReabilitaçãoSão PauloSPBrasilUniversidade Nove de Julho - Programa de Pós-Graduação em Ciências da Reabilitação, São Paulo, SP – Brasil; 3 Penn State College of MedicineHersheyEUAPenn State College of Medicine, Hershey – EUA; 4 Northumbria UniversityNewcaslte Upon TyneReino UnidoNorthumbria University, Newcaslte Upon Tyne – Reino Unido

**Keywords:** Teste de Caminhada/métodos, Doença Arterial Periférica, Esforço Físico, Exercício, Claudicação Intermitente, Capacidade Vital/fisiologia

## Abstract

**Fundamento:**

a caminhada não supervisionada em solo tem sido indicada para pacientes com doença arterial periférica (DAP) sintomática. No entanto, a magnitude do esforço exigido por essa atividade e as características dos pacientes que a praticam com mais intensidade não estão claras.

**Objetivos:**

determinar se a caminhada em solo excede o limiar ventilatório (LV), um reconhecido marcador de intensidade de exercício, em pacientes com DAP sintomática.

**Métodos:**

Foram recrutados 70 pacientes (61,4% do sexo masculino e com idade entre 40 e 85 anos) com DAP sintomática. Os pacientes realizaram um teste ergométrico em esteira para definir o LV. Em seguida, foram submetidos ao teste de caminhada de 6 minutos para determinar o alcance do LV durante deambulação no solo. Realizou-se regressão logística múltipla para identificar preditores de LV durante o teste de caminhada de 6 minutos, e o valor de p<0,05 foi considerado significativo para todas as análises.

**Resultados:**

Ao todo, 60% dos pacientes atingiram o LV durante o teste de caminhada de 6 minutos. Mulheres (OR = 0,18 e IC95% = 0,05 a 0,64) e pacientes com mais aptidão cardiorrespiratória (OR = 0,56 e IC 95% = 0,40 a 0,77) tiveram menor probabilidade de chegar ao LV durante a caminhada em solo em comparação a homens e pacientes com menos aptidão cardiorrespiratória, respectivamente.

**Conclusão:**

Mais da metade dos pacientes com DAP sintomática alcançou o LV durante o teste de caminhada de 6 minutos. Mulheres e pacientes com mais aptidão cardiorrespiratória têm menos probabilidade de chegar ao LV durante o teste de caminhada de 6 minutos, o que indica que a caminhada no solo pode ser mais intensa para esse grupo. Isso deve ser considerado ao se prescreverem exercícios de caminhada em solo para esses pacientes. (Arq Bras Cardiol. 2020; 114(3):486-492)

## Introdução

A doença arterial periférica (DAP) afeta aproximadamente 12% dos adultos com idade mais avançada nos Estados Unidos^1^ e 21,6% da população idosa no Brasil.^2^ Pacientes com DAP sintomática (claudicação intermitente) têm sua capacidade de deambulação prejudicada,^3^ menos força muscular^4,5^ e várias comorbidades.^6^ Além disso, pacientes com DAP sintomática apresentam baixa aptidão cardiorrespiratória, evidenciada pelo menor consumo de pico de oxigênio (VO_2_) e pior economia de marcha do que seus controles da mesma idade.^7^ Portanto, nesses pacientes, a caminhada realizada durante atividades rotineiras diárias já é realizada em intensidade relativamente mais alta em comparação aos controles da mesma idade.

O limiar ventilatório (LV) é um importante marcador de intensidade de exercício. LV mais alto indica que os pacientes podem sustentar um aumento no metabolismo anaeróbico durante o esforço.^8^ Em pacientes com DAP sintomática, um LV mais baixo está associado a menor tolerância à caminhada e maior gravidade da doença.^9,10^ Além disso, o LV tem mais probabilidade de ser alcançado antes do início da dor de claudicação.^11,12^

A caminhada em solo tem sido amplamente utilizada para avaliar o comprometimento da deambulação em pacientes com DAP através de um teste de caminhada de 6 minutos, pois trata-se do principal resultado clínico nesse grupo.^13^ Recentemente, também tem sido utilizado em programas de exercícios em casa. No entanto, a intensidade em que a caminhada em solo é realizada por pacientes com DAP é desconhecida. Do ponto de vista prático, compreender a magnitude do esforço no teste de caminhada de 6 minutos pode dar suporte ao uso dessa atividade como modalidade de exercício para pacientes com DAP. Assim, o objetivo deste estudo foi descrever a intensidade do teste de caminhada de 6 minutos de acordo com o LV em pacientes com DAP sintomática. Também foram analisados os preditores de alcance do LV durante o teste.

## Methods

Os procedimentos deste estudo foram aprovados pelo Conselho de Revisão Institucional do Centro de Ciências da Saúde da Universidade de Oklahoma (*Institutional Review Board, University of Oklahoma Health Sciences Center*, protocolo nº 2337). Foi obtido um consentimento livre e informado por escrito de cada paciente antes de sua participação.

### Recrutamento e pacientes

Pacientes com DAP classificados como Rutherford Grau I e Categoria 1 a 3 foram avaliados no Centro de Pesquisa Clínica do Centro de Ciências da Saúde da Universidade de Oklahoma. Os pacientes chegaram em jejum, mas foram autorizados a tomar seus medicamentos habituais. Todos foram recrutados por encaminhamento das clínicas vasculares do Centro de Ciências da Saúde, bem como por anúncios em jornais para possível inscrição em um estudo ligado a exercícios físicos.^14,15^ No entanto, os pacientes foram incluídos no estudo se estivessem totalmente dentro dos seguintes critérios: (a) teste ergométrico em esteira com limitações devido a sintomas de claudicação intermitente e (b) índice tornozelo-braquial (ITB) ≥ 0,90 em repouso ou ITB ≥ 0,73 após o exercício.^1^

Os pacientes excluídos da amostra correspondiam a algum dos seguintes critérios: (a) impossibilidade de obtenção de ITB devido a vasos não compressíveis (ITB ≥ 1,40); (b) DAP assintomática determinada a partir de seu histórico médico e verificada no teste ergométrico em esteira, (c) tolerância ao exercício durante o teste ergométrico em esteira, limitado por outros fatores que não os sintomas de claudicação (por exemplo, alterações eletrocardiográficas clinicamente significativas durante o exercício, indicativas de isquemia miocárdica, dispneia, pressão arterial mal controlada), (d) falha em alcançar o LV durante o exercício na esteira (e); não conclusão do teste de caminhada de 6 minutos sem paradas e (f) não conclusão do teste dentro de três semanas.

### Desenho do estudo

**Figura 1:**
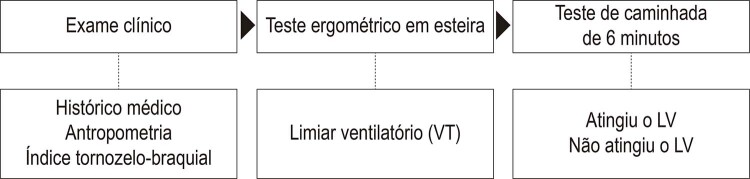
– Desenho do estudo.

Este estudo foi dividido em três etapas: 1) exame clínico, 2) teste ergométrico na esteira e 3) teste de caminhada de 6 minutos. A etapa 1 incluiu avaliações para histórico médico, antropometria e ITB. Na etapa 2, os pacientes realizaram um teste cardiopulmonar progressivo em esteira rolante até a máxima dor de claudicação, a fim de alcançar o LV. Na etapa 3, foi aplicado o teste de caminhada de 6 minutos, com o objetivo de identificar os pacientes que não atingiram e que atingiram o LV (Figura 1). Os detalhes de todas as avaliações estão descritos abaixo.

### Histórico Médico e Antropometria

Informações demográficas, de altura, peso, índice de massa corporal, circunferência da cintura, histórico de claudicação, exame físico e condições comórbidas (osteoartrite, obesidade, hipertensão, diabetes, dislipidemia, síndrome metabólica e doença cardíaca) foram avaliadas no início do estudo por um médico. A obesidade foi definida como índice de massa corporal >30 kg/m^2^.^16^ A hipertensão foi definida como pressão arterial sistólica ≥140 mmHg ou diastólica ≥ 90 mmHg ou uso de medicamentos anti-hipertensivos.^16^ O diabetes foi definido como glicemia de jejum ≥ 126 mg/dl ou uso de medicação hipoglicêmica.^17^ Dislipidemia foi definida como triglicerídeos ≥ 150 mg/dl, LDL-C ≥ 130 mg/dl, colesterol total ≥ 200 mg/dl ou HDL-C ≤ 40 mg/dl (homens) e ≤ 50 mg/dl (mulheres) ou uso de medicamentos hipolipemiantes.^18^ A síndrome metabólica foi definida por três ou mais dos seguintes componentes: (1) obesidade abdominal (circunferência da cintura >102 cm em homens e >88 cm em mulheres), (2) triglicerídeos elevados (>150 mg/dl), (3) HDL-C reduzido (<40 mg/dl em homens e <50 mg/dl em mulheres), (4) pressão arterial elevada (>130/85 mmHg) e (5) glicose em jejum elevada (> 110 mg/dl), bem como diagnóstico de diabetes.^19^

### Índice tornozelo-braquial

O ITB foi obtido após 10 minutos de repouso em decúbito dorsal, medindo-se a pressão arterial sistólica do tornozelo e braquial pela técnica Doppler na artéria braquial e nas artérias pedais tibial e dorsal posterior. O valor mais alto entre as duas medidas da pressão arterial de cada perna foi registrado e a perna com o menor ITB foi utilizada nas análises, como descrito anteriormente.^20^

### Teste ergométrico em esteira

Um teste em esteira graduada foi utilizado para obter o LV e avaliar a capacidade de caminhar. Os pacientes realizaram um teste cardiopulmonar progressivo em esteira até dor máxima de claudicação, como descrito anteriormente.^21^ O teste começou a 2 mph com classificação de 0%, e a carga foi aumentada em 2% a cada 2 minutos. Todos os pacientes foram informados do protocolo do teste antes de serem submetidos a ele. O consumo de oxigênio (VO_2_) foi medido continuamente por um calorímetro portátil (Medical Graphics Corp., St Paul, MN), e médias de 30s foram aplicadas para análise. O LV foi detectado visualmente por dois avaliadores experientes e definido como um aumento não linear no quociente respiratório, produção de dióxido de carbono e ventilação, bem como o aumento da pressão expiratória final. Foram analisadas as seguintes variáveis: captação de oxigênio (VO_2_), emissão de dióxido de carbono (VCO_2_), equivalente ventilatório (EV), equivalente ventilatório para O_2_ (EV/VO_2_), equivalente ventilatório para CO_2_ (EV/VO_2_), pressão expiratória final (PEFO_2_) e pressões parciais de dióxido de carbono (PEFCO_2_), e razão de troca respiratória, como descrito anteriormente.^22^ Um terceiro pesquisador comparou os resultados para verificar possíveis discrepâncias na determinação do LV entre avaliadores. Nesse caso, o LV foi novamente determinado pelos avaliadores e o terceiro avaliador fez a final. Considerou-se que os pacientes que não apresentavam nenhum desses parâmetros respiratórios durante o teste cardiopulmonar progressivo em esteira não atingiram o LV e, portanto, foram excluídos da amostra.

### Medições de claudicação e captação de pico de oxigênio

O momento do início da claudicação foi definido como o momento da caminhada em que o paciente experimentou dor nas pernas pela primeira vez durante o teste na esteira e o pico de caminhada foi definido como o momento da caminhada em que os pacientes não puderam continuar andando devido à dor nas pernas. O pico do VO_2_ foi definido como a janela de 30 segundos com o maior VO_2_ alcançado durante o teste em esteira. Com esses procedimentos, os coeficientes de confiabilidade intraclass e teste-reteste são r = 0,89 para o momento de início da claudicação e r = 0,93 para o pico de caminhada.^24^

### Teste de caminhada de 6 minutos

Um técnico treinado administrou o teste de caminhada de 6 minutos, realizado em um corredor de 30 metros de comprimento. Os indivíduos foram instruídos a caminhar o máximo de voltas possível ao redor dos cones, carregando uma unidade de captação de oxigênio leve (0,8 kg) portátil (COSMED K4 b^2^, COSMED USA, Inc, Chicago, IL), que media continuamente a captação de oxigênio por calorimetria indireta. O técnico estava cego para os resultados do LV, e o teste foi realizado de acordo com as instruções padronizadas, como descrito anteriormente.^23^ O VO_2_ foi obtido respiração por respiração e, em seguida, foi calculada a média a cada minuto durante o teste, o que permitiu identificar os pacientes que alcançaram o LV. Para isso, os pacientes deveriam ter completado o teste sem realizar paradas após os sintomas de claudicação intermitente. Assim, os pacientes foram divididos em dois grupos: os que não alcançaram e os que alcançaram o LV durante a caminhada em solo.

### Análise estatística

As análises estatísticas foram realizadas no software Statistical Package for the Social Sciences – SPSS/PASW versão 20 (IBM Corp, Nova York, EUA). A normalidade dos dados foi verificada pelo teste de Shapiro-Wilk. As variáveis contínuas foram resumidas em média e desvio-padrão, enquanto as variáveis categóricas foram expressas em frequência relativa. Os pacientes foram agrupados de acordo com o LV alcançado ou não, e as características clínicas entre os grupos foram comparadas pelo teste t independente para variáveis contínuas e pelo teste do qui-quadrado para variáveis categóricas.

Regressão logística múltipla foi realizada para identificar se dados demográficos, fatores de risco cardiovascular, condições comórbidas, ITB e capacidade de caminhar são preditores de alcance do LV durante o teste de caminhada de 6 minutos. Para tanto, foram utilizadas técnicas *stepwise backward* para inserir covariáveis no modelo, utilizando apenas variáveis com p < 0,30 nas análises bivariadas. Na regressão múltipla, apenas variáveis com p < 0,05 permaneceram no modelo final. O teste de Hosmer-Lemeshow foi usado para avaliar a adequação geral do modelo. O nível de significância foi estabelecido em p < 0,05 para todas as análises.

## Resultados

Cento e trinta e três pacientes realizaram o teste de caminhada de 6 minutos. Entre eles, 63 pararam durante o teste devido a sintomas de claudicação e foram excluídos da análise. Dentre os 70 pacientes que não pararam durante o teste, o LV foi alcançado durante o teste de caminhada de 6 minutos por 42 deles (60%) e não foi alcançado por 28 deles (40%). A Tabela 1 mostra a comparação das características clínicas dos pacientes que atingiram e que não atingiram o LV durante a caminhada em solo. O VO_2_ no LV obtido durante o teste em esteira foi maior nos pacientes que não alcançaram o LV durante o teste de caminhada de 6 minutos em comparação aos pacientes que alcançaram (p < 0,05). Além disso, o índice tornozelo-braquial foi maior nos pacientes que não alcançaram o LV em comparação aos pacientes que alcançaram (p < 0,05).

**Tabela 1:** – Características dos pacientes com claudicação intermitente incluídos no estudo

Variáveis	Não atingiu o LV (n = 28)	Atingiu o LV (n = 42)	p
Idade, em anos	66,1 ± 9,9	66,9 ± 10,2	0,745
Índice de massa corporal, kg^-1^m^2^	29,9 ± 6,0	29,0 ± 5,6	0,486
Índice Tornozelo-Braço	0,85 ± ,21	0,71 ± ,21	0,013
Início da claudicação, em segundos	297 ± 192	271 ± 191	0,572
Pico de caminhada, em segundos	576 ± 266	541 ± 219	0,542
Teste de 6 minutos, distância sem dor, em metros	189 ± 144	214 ± 96	0,417
Teste de caminhada de 6 minutos, em metros	382 ± 73	399 ± 67	0,332
VO_2_ no LV, mL.kg^-1^.min^-1^	12,0 ± 2,4	10,1 ± 1,9	< 0,001
Pico de VO_2_, mL.kg^-1^.min^-1^	13,9 ± 3,7	13,5 ± 3,4	0,627
Sexo, % de mulheres	52	48	0,109
*Diabetes mellitus*, % sim	46	54	0,419
Hipertensão, % sim	41	59	0,789
Dislipidemia, % sim	38	62	0,436
Doença arterial coronariana, % sim	13	88	0,093
DPOC, % sim	53	47	0,211

LV: limiar ventilatório; VO_2:_ Captação de oxigênio; DPOC: doença pulmonar obstrutiva crônica.

A Tabela 2 mostra os preditores para atingir o LV durante o teste de caminhada de 6 minutos. As mulheres foram menos propensas a atingir o LV durante a caminhada em solo do que os homens (p < 0,05). Além disso, pacientes com maior VO_2_ no LV apresentaram menor probabilidade de chegar ao LV durante a caminhada em solo (p < 0,05).

**Tabela 2:** – Modelo de regressão logística múltipla que prediz o limiar ventilatório alcançado durante o teste de caminhada de 6 minutos em pacientes com claudicação intermitente

Variável dependente	Variáveis independentes	β (EP)	OR	IC95%	p
Alcançou LV	Sexo, masculino = referência	-1,72 (0,65)	0,18	0,05 – 0,64	0,008
	Captação de oxigênio no LT, mL.kg^-1^.min^-1^	- 0,58 (0,17)	0,56	0,40 – 0,77	< 0,001

LV: limiar ventilatório. β (EP): Coeficiente de regressão (padrão de erro); OR: Odds Ratio. IC95%: intervalo de confiança de 95%. Teste de Hosmer-Lemeshow: χ^2^ = 9,607, p = 0,298.

A Tabela 3 mostra as comparações por sexo. A prevalência de obesidade foi maior e a aptidão cardiorrespiratória foi menor nas mulheres em comparação aos homens (p < 0,05). O pico de VO_2 _no teste de caminhada de 6 minutos e no teste em esteira foi maior nos homens do que nas mulheres (p < 0,05).

**Tabela 3:** – Comparação dos parâmetros clínicos de claudicação intermitente entre homens e mulheres incluídos no estudo

Variáveis	Mulheres (n = 28)	Homens (n = 43)	p
Idade, em anos	64,9 ± 9,5	67,6 ± 10,3	0,265
Índice de massa corporal, kg^-1^m^2^	31,1 ± 6,6	28,3 ± 5,0	0,044
Índice Tornozelo-Braço	0,80 ± ,23	0,75 ± ,22	0,258
Início da claudicação, em segundos	241 ± 164	306 ± 203	0,330
Pico de caminhada, em segundos	507 ± 196	585 ± 256	0,180
VO_2_ no LV, mL.kg^-1^.min^-1^	10,3 ± 2,3	11,3 ± 2,2	0,035
Pico de VO_2_ no teste ergométrico em esteira, mL.kg^-1^.min^-1^	12,0 ± 2,9	14,7 ± 3,4	0,001
Pico do VO_2_ no TC6M, mL.kg^-1^.min^-1^	11,1 ± 3,0	12,5 ± 2,1	0,034

TC6M: teste de caminhada de 6 minutos.

## Discussão

Os principais achados do estudo foram: a) 60% dos pacientes com DAP sintomática atingiram o LV durante o teste de caminhada de 6 minutos e b) mulheres e pacientes com maior VO_2_ no LV durante o teste na esteira foram menos propensos a atingir o LV no teste de caminhada de 6 minutos.

O LV é definido como a intensidade do exercício acima da qual a predominância metabólica muda de aeróbica para anaeróbica,^8^ fornecendo informações sobre a capacidade aeróbica durante o exercício. Em pacientes com DAP sintomática, o LV está associado à tolerância à caminhada e à gravidade da doença.^9,10^ Neste estudo, 60% dos pacientes alcançaram o LV no teste de caminhada de 6 minutos, indicando que, para a maioria dos pacientes com DAP sintomática, a caminhada em solo é um exercício de intensidade relativamente alta. Isso poderia explicar parcialmente os níveis mais baixos de atividade física diária e o maior tempo sedentário desses pacientes.^25,26^ Portanto, o nível de intensidade da caminhada em solo na maioria dos pacientes com DAP exige um esforço bastante alto, o que sugere que este exercício tem potencial para melhorar a capacidade funcional desse grupo e, portanto, dá suporte ao uso de programas para melhorar aptidão cardiorrespiratória em casa

Por outro lado, quase 40% dos pacientes não atingiram o LV no teste de caminhada de 6 minutos. A hipótese mais plausível para a porção dos pacientes com DAP que não alcançaram o LV foi de que a caminhada em solo não era intensa o suficiente para induzir a isso. Essa hipótese encontra suporte no fato de que pacientes com melhor aptidão cardiorrespiratória foram menos propensos a alcançar o LV durante a caminhada em solo.

As mulheres tiveram menos probabilidade de exceder o LV durante o teste de caminhada de 6 minutos do que os homens, indicando realização em menor intensidade relativa pelas mulheres do que pelos homens. Isso é surpreendente dado que estudos anteriores^27,28^ demonstraram que mulheres com DAP sintomática apresentam menor capacidade de deambulação,^29,30^ são menos ativas fisicamente^29,30^ e relatam mais obstáculos à prática de atividade física em comparação aos homens.^31^ Além disso, as mulheres têm mais características adversas nos músculos da panturrilha e menor pico de VO_2_ que os homens.^32^

Algumas mensagens práticas podem ser retiradas deste estudo. O teste de caminhada de 6 minutos é mais difícil para homens e pacientes com baixa aptidão cardiorrespiratória. Portanto, leva a uma intensidade maior de exercício nesses pacientes. Além disso, recomenda-se que a intensidade do treinamento físico seja realizada acima do LV para melhorar a função cardiovascular em pacientes cardíacos e idosos.^33,34^ Considerando que o teste de caminhada de 6 minutos simula uma caminhada em solo, os resultados atuais dão suporte ao seu uso como modalidade de exercício para aumentar a atividade física diária e a aptidão cardiorrespiratória em homens e em pacientes com baixa aptidão cardiorrespiratória. No entanto, em mulheres e pacientes com maior aptidão cardiorrespiratória, a caminhada em solo pode não ser suficiente para melhorar os níveis de atividade e aptidão.

O desenho transversal deste estudo é uma limitação, pois nenhuma causalidade pode ser inferida. Pacientes com doença cardíaca grave e DAP assintomática ou DAP mais grave que claudicação foram excluídos na triagem; portanto, os resultados podem ser estendidos apenas à nossa amostra atual de pacientes com claudicação. Como não conseguimos identificar com precisão o LV em pacientes que fizeram paradas durante o teste de caminhada de 6 minutos, a generalização também é restrita a eles. Além disso, para detectar com precisão o LV no teste de caminhada de 6 minutos, incluímos apenas pacientes que não pararam durante a sua execução. Esses achados também são limitados pelo tamanho relativamente pequeno da amostra, principalmente quando se trata de pacientes que não atingiram o LV.

## Conclusão

Mais da metade dos pacientes com DAP sintomática alcançou o LV durante o teste de caminhada de 6 minutos. Homens e pacientes com baixa aptidão cardiorrespiratória têm maior probabilidade de chegar ao LV durante o teste de caminhada de 6 minutos.
