# Supplementary material for: Intensidade de Exercício durante o Teste de Caminhada de 6 Minutos em Pacientes com Doença Arterial Periférica
Source: Arq Bras Cardiol. 2020 Apr 6;114(3):486–92. [Article in Portuguese] doi: 10.36660/abc.20190053 (PMC7792737; doi:10.36660/abc.20190053)
Supplement: Translation

# Translation: Exercise Intensity during 6-Minute Walk Test in Patients with Peripheral Artery Disease

**Authors:** Breno Quintella Farah, Raphael Mendes Ritti-Dias, Polly Montgomery, Gabriel Grizzo Cucato, Andrew Gardner

**Affiliations:** 1 BrazilUniversidade Federal Rural de Pernambuco, Recife, PE – Brazil; 2 BrazilUniversidade Nove de Julho - Programa de Pós-Graduação em Ciências da Reabilitação, São Paulo, SP – Brazil; 3 USAPenn State College of Medicine, Hershey – USA; 4 Reino UnidoNorthumbria University, Newcaslte Upon Tyne – Reino Unido

**Keywords:** Walk Test/methods, Peripheral Arterial Disease/complications, Physical Exertion, Exercise, Intermittent Claudication, Vital Capacity/physiology

## Abstract

**Background:**

Non-supervised ground walking has been recommended for patients with symptomatic peripheral artery disease (PAD). However, the magnitude of the effort required by this activity and the characteristics of patients whose ground walking is more intense are unclear.

**Objectives:**

To determine whether ground walking exceeds the ventilatory threshold (VT), a recognized marker of exercise intensity, in patients with symptomatic PAD.

**Methods:**

Seventy patients (61.4% male and aged 40 to 85 years old) with symptomatic PAD were recruited. Patients performed a graded treadmill test for VT determination. Then, they were submitted to a 6-minute walk test so the achievement of VT during ground ambulation could be identified. Multiple logistic regression was conducted to identify predictors of VT achievement during the 6-minute walk test. The significance level was set at p < 0.05 for all analyses.

**Results:**

Sixty percent of patients achieved VT during the 6-minute walk test. Women (OR = 0.18 and 95%CI = 0.05 to 0.64) and patients with higher cardiorespiratory fitness (OR = 0.56 and 95%CI = 0.40 to 0.77) were less likely to achieve VT during ground walking compared to men and patients with lower cardiorespiratory fitness, respectively.

**Conclusion:**

More than half of patients with symptomatic PAD achieved VT during the 6-minute walk test. Women and patients with higher cardiorespiratory fitness are less likely to achieve VT during the 6-minute walk test, which indicates that ground walking may be more intense for this group. This should be considered when prescribing ground walking exercise for these patients. (Arq Bras Cardiol. 2020; 114(3):486-492)

## Introduction

Peripheral artery disease (PAD) affects approximately 12% of older adults in the United States^1^ and 21.6% of the elderly population in Brazil.^2^ Patients with symptomatic PAD (intermittent claudication) have impaired walking capacity,^3^ lower muscular strength,^4,5^ and several comorbid conditions.^6^ In addition, patients with symptomatic PAD present poor cardiorespiratory fitness evidenced by lower peak oxygen consumption (VO_2_) and worse walking economy than age-matched controls.^7^ Therefore, in these patients, walking performed during everyday activities are done at relatively higher intensities than in age-matched controls.

Ventilatory threshold (VT) is an important marker of exercise intensity. Higher VT indicates that patients can sustain an increase in anaerobic metabolism during exercise.^8^ In symptomatic PAD patients, lower VT is associated with lower walking tolerance and greater disease severit.^9,10^ In addition, VT is most likely to be achieved before the onset of claudication pain.^11,12^

Ground walking have been widely used to assess walking impairment in PAD patients through a 6-minute walk test, as this is a main clinical outcome in this group.^13^ Recently, it has also been used in home-based exercise programs. However, the intensity in which ground walking is performed by patients with PAD is unknown. From a practical point of view, understanding the magnitude of effort in the 6-minute walk test might support the use of over-ground as an exercise modality in PAD patients. Thus, the purpose of this study was to describe the intensity of the 6-minute walk test according to VT in patients with symptomatic PAD. We also analyzed the predictors of the achievement of VT during the 6-minute test.

## Methods

The procedures of this study were approved by the Institutional Review Board at the University of Oklahoma Health Sciences Center (protocol #2337). A written informed consent was obtained from each patient prior to their participation.

### Recruitment and Patients

PAD patients classified as Rutherford Grade I and Category 1 to 3 were evaluated at the Clinical Research Center at the University of Oklahoma Health Sciences Center. Patients arrived fasted, but were permitted to take their usual medications. Patients were recruited by referrals from the Health Sciences Center vascular clinics, as well as by newspaper advertisements for possible enrollment into an exercise study.^14,15^ However, patients were included in the study if they fully met the following criteria: (a) graded treadmill test limited by intermittent claudication symptoms and (b) an ankle brachial index (ABI) ≤ 0.90 at rest, or an ABI ≤ 0.73 after exercise.^1^

Patients were excluded if they met any of the following criteria: (a) inability to obtain an ABI measure due to non-compressible vessels (ABI ≥ 1.40), (b) asymptomatic PAD determined from their medical history and verified upon the graded treadmill test, (c) exercise tolerance during progressive treadmill test limited by factors other than claudication symptoms (e.g. clinically significant electrocardiographic changes during exercise indicative of myocardial ischemia, dyspnea, poorly controlled blood pressure), (d) failure to achieve VT during treadmill exercise, (e) inability to complete the 6-minute walk test without stopping, and (f) failure to complete the testing within three weeks.

### Study Design

This study was divided into three steps: 1) clinical examination, 2) graded treadmill test, and, 3) 6-minute walk test. Step 1 included evaluations for medical history, anthropometry, and ankle-brachial index. During step 2, patients performed a progressive graded cardiopulmonary treadmill test until maximal claudication pain, in order to obtain the VT. In step 3, the 6-minute walk test was applied aiming to identify the patients who did not and those who did achieve VT (Figure 1). The details of all evaluations are described below.

**Figure 1:** – Study design.

### Medical History and Anthropometry

Demographic information, height, weight, body mass index, waist circumference, claudication history, physical examination and comorbid conditions (osteoarthritis, obesity, hypertension, diabetes, dyslipidemia, metabolic syndrome and heart disease) were assessed at the beginning of the study by a physician. Obesity was defined as body mass index > 30 kg/m^2^.^16^ Hypertension was defined as systolic blood pressure ≥ 140 mmHg or diastolic blood pressure ≥ 90 mmHg, or use of anti-hypertensive medication.^16^ Diabetes was defined as fasting blood glucose ≥ 126 mg/dl, or use of hypoglycemic medication.^17^ Dyslipidemia was defined as triglycerides ≥ 150 mg/dl, LDL-C ≥ 130 mg/dl, total cholesterol ≥ 200 mg/dl or HDL-C ≤ 40 mg/dl (men) and ≤ 50 mg/dl (women), or use of lipid-lowering medication.^18^ Metabolic syndrome was defined as three or more of the following components: (1) abdominal obesity (waist circumference >102 cm in men and > 88 cm in women), (2) elevated triglycerides (>150 mg/dl), (3) reduced HDL-C (< 40 mg/dl in men and < 50 mg/dl in women), (4) elevated blood pressure (> 130/85 mmHg), and (5) elevated fasting glucose (> 110 mg/dl), as well as diagnosis of diabetes.^19^

### Ankle Brachial Index

ABI was obtained after 10 minutes of supine rest by measuring the ankle and brachial systolic blood pressure using Doppler technique in the brachial artery and both posterior tibial and dorsalis pedis arteries. The highest value between the two measurements of arterial pressure from each leg was recorded, and the leg yielding the lowest ABI was used in the analyses, as previously described.^20^

### Graded Treadmill Test

A graded treadmill test was used to obtain the VT and to assess walking capacity. Patients performed a progressive graded cardiopulmonary treadmill test until maximal claudication pain, as previously described.^21^ The test started at 2 mph with 0% grade and the workload was increased 2% every 2 minutes. All patients were informed of the test protocol before being submitted to it. Oxygen consumption (VO_2_) was continuously measured by a metabolic cart (Medical Graphics Corp., St Paul, MN), and averages of 30s were applied for analysis. The VT was visually detected by two experienced evaluators and defined as a nonlinear increase in respiratory quotient, carbon dioxide production and ventilation, as well as the increase in end-tidal oxygen pressure. The following variables were analyzed: oxygen uptake (VO_2_), carbon dioxide output (VCO_2_), ventilatory equivalent (VE), ventilatory equivalent for O_2_ (VE/VO_2_), ventilatory equivalent for CO_2 _(VE/VO_2_), end-tidal oxygen (PETO_2_) and carbon dioxide partial pressures (PETCO_2_), and respiratory exchange ratio, as previously described.^22^ A third researcher compared the results to check possible discrepancies in the determination of VT between evaluators. In this case, the determination of VT was repeated by both evaluators and the third evaluator made the final determination. Patients not presenting any of these respiratory parameters during the progressive graded cardiopulmonary treadmill test were considered to not have achieved the VT and were therefore excluded from the sample.

### Claudication Measurements and Peak Oxygen Uptake

The claudication onset time was defined as the walking time at which the patient first experienced leg pain during the treadmill test, and the peak walking time was defined as the walking time at which the patients could not continue walking due the leg pain. VO_2 _peak was defined as the 30-second window with the highest VO_2 _achieved during the treadmill test. Using these procedures, the test-retest intra-class reliability coefficients are r = 0.89 for claudication onset time and r = 0.93 for peak walking time.^24^

### 6-minute Walk Test

A trained technician administered the 6-minute walk test which was conducted in a 30-meter long corridor. Subjects were instructed to walk as many laps around the cones as possible while bearing a light weight (0.8 kg), portable oxygen uptake unit (COSMED K4 b^2^, COSMED USA, Inc, Chicago, IL) which continuously measured oxygen uptake via indirect calorimetry. The technician was blinded to the VT results, and the test was performed following the standardized instructions, as previously described.^23^ VO_2 _was obtained breath-by-breath and then averaged per each minute during the test, which allowed for the identification of patients who achieved VT. For this, patients were supposed to have completed the test without stopping after intermittent claudication symptoms. Thus, patients were divided into two groups: those who did not achieve VT and those who did achieve VT during ground walking.

### Statistical Analysis

All statistical analyses were made in the Statistical Package for the Social Sciences software – SPSS/PASW version 20 (IBM Corp, New York, USA). Normality of data was checked by the Shapiro-Wilk test. Continuous variables were summarized as mean and standard deviation, whereas categorical variables were expressed as relative frequency. Patients were grouped according to whether or not they achieved VT, and the clinical characteristics between groups were compared by the independent t-test for continuous variables and the chi-square test for categorical variables.

Multiple logistic regression was conducted to identify whether demographic data, cardiovascular risk factors, comorbid conditions, ABI and walking capacity are predictors of achieving VT during the 6-minute walk test. To this end, stepwise backward techniques were used to enter covariates into the model using only variables with p < 0.30 in the bivariate analyses. In the multiple regression, only variables with p < 0.05 remained in the final model. The Hosmer-Lemeshow test was used to assess the model’s goodness-of-fit. The significance level was set at p < 0.05 for all analyses.

## Results

One hundred and thirty-three patients performed the 6-minute walk test. Among them, 63 stopped during the test due to claudication symptoms and were excluded from the analysis. Among the 70 patients who did not stop during the test, the VT was achieved during the 6-minute walk test by 42 patients (60%) and was not achieved by 28 patients (40%). Table 1 shows the comparison of clinical characteristics of patients who achieved and who did not achieve the VT during 6-minute walk test. VO_2_ at VT obtained during the treadmill test was higher in patients who did not achieve VT during the 6-minute walk test in comparison with patients who achieved it (p < 0.05). Moreover, the ankle brachial index was higher in patients who did not achieve VT in comparison with patients who did (p < 0.05).

**Table 1:** – Characteristics of patients with intermittent claudication included in the study

Variables	Did not achieve VT (n = 28)	Achieved VT (n = 42)	p
Age, years	66.1 ± 9.9	66.9 ± 10.2	0.745
Body mass index, kg^-1^m^2^	29.9 ± 6.0	29.0 ± 5.6	0.486
Ankle brachial index	0.85 ± .21	0.71 ± .21	0.013
Claudication onset time, seconds	297 ± 192	271 ± 191	0.572
Peak walking time, seconds	576 ± 266	541 ± 219	0.542
Six-minute pain-free distance, meters	189 ± 144	214 ± 96	0.417
Six-minute walk test, meters	382 ± 73	399 ± 67	0.332
VO_2_ at VT, mL.kg^-1^.min^-1^	12.0 ± 2.4	10.1 ± 1.9	< 0.001
VO_2 _peak, mL.kg^-1^.min^-1^	13.9 ± 3.7	13.5 ± 3.4	0.627
Sex, % women	52	48	0.109
Diabetes mellitus, % yes	46	54	0.419
Hypertension, % yes	41	59	0.789
Dyslipidemia, % yes	38	62	0.436
Coronary artery disease, % yes	13	88	0.093
COPD, % yes	53	47	0.211

VT: ventilatory threshold; VO_2:_ oxygen uptake; COPD: chronic obstructive pulmonary disease.

Table 2 shows the predictors to achieve the VT during the 6-minute walk test. Women were less likely to achieve VT during 6-minute walk test than men (p < 0.05). Moreover, patients with higher VO_2_ at VT were less likely to achieve the VT during 6-minute walk test (p < 0.05).

**Table 2:** – Multiple logistic regression model predicting achieved ventilatory threshold during the 6-minute walk test in patients with intermittent claudication

Dependent variable	Independent variables	β (EP)	OR	95%CI	p
Achieved VT	Sex, men = reference	-1.72 (0.65)	0.18	0.05 – 0.64	0.008
	Oxygen uptake at VT, mL.kg^-1^.min^-1^	- 0.58 (0.17)	0.56	0.40 – 0.77	< 0.001

VT: ventilatory threshold; β (EP): Regression coefficient (error-standard); OR: odds-ratio. 95% CI: 95% confidence interval. Hosmer-Lemeshow test: χ^2^ = 9.607, p = 0.298.

Table 3 shows the comparisons by sex. In women, the prevalence of obesity was higher and cardiorespiratory fitness was lower as compared to men (p < 0.05). VO_2 _peak in both the 6-minute walk test and the treadmill test was higher in men than in women (p < 0.05).

**Table 3:** – Comparison of clinical parameters of intermittent claudication between men and women included in the study

Variables	Women (n = 28)	Men (n = 43)	p
Age, years	64.9 ± 9.5	67.6 ± 10.3	0.265
Body mass index, kg^-1^m^2^	31.1 ± 6.6	28.3 ± 5.0	0.044
Ankle brachial index	0.80 ± .23	0.75 ± .22	0.258
Claudication onset time, seconds	241 ± 164	306 ± 203	0.330
Peak walking time, seconds	507 ± 196	585 ± 256	0.180
VO_2_ at VT, mL.kg^-1^.min^-1^	10.3 ± 2.3	11.3 ± 2.2	0.035
VO_2 _peak in treadmill test, mL.kg^-1^.min^-1^	12.0 ± 2.9	14.7 ± 3.4	0.001
VO_2 _peak in 6-MWT, mL.kg^-1^.min^-1^	11.1 ± 3.0	12.5 ± 2.1	0.034

6-MWT: 6-minute walk test.

## Discussion

The main findings of the study were: a) 60% of symptomatic PAD patients did achieve the VT during 6 minute-walk test, and b) women and patients with higher VO_2_ at VT obtained during the treadmill test were less likely to achieve VT in the 6-minute-walk test.

VT is defined as the exercise intensity above which metabolic predominance changes from aerobic to anaerobic,^8^ providing information about aerobic capacity during exercise. In patients with symptomatic PAD, VT have been associated with walking tolerance and disease severity.^9,10^ In this study, 60% of patients achieved VT in the 6-minute walk test, indicating that for most patients with symptomatic PAD this ground walking is a relatively high-intensity exercise. This could partially explain the lower daily physical activity levels and higher time spent in sedentary behavior in these patients.^25,26^ Therefore, the intensity in which ground walking is performed by most patients with PAD demands a fairly high effort, which suggests that this exercise has potential to improve functional capacity of patients with PAD and, therefore, endorses the use of home-based programs to improve cardiorespiratory fitness.

On the other hand, almost 40% of the patients did not achieve VT in the 6-minute walk test. The most plausible hypothesis for part of PAD patients not achieving VT was that the ground walking was not intense enough to elicit VT achievement. This hypothesis is corroborated by the fact that patients with higher cardiorespiratory fitness were less likely to achieve the VT during ground walking.

Women were less likely to exceed VT during the 6-minute walk test than men, indicating that ground walking is performed at a lower relative intensity by women than by men. This is surprising given previous studies^27,28^ have shown that women with symptomatic PAD have lower walking capacity,^29,30^ are less physically active^29,30^ and report more barriers to practicing physical activity compared to men.^31^ In addition, women present more adverse calf muscle characteristics and lower VO_2 _peak than men.^32^

Some practical messages can be draw from this study. The 6-minute walk test is harder for men and patients with low cardiorespiratory fitness. Is recommended that exercise training intensity should be performed above VT in order to improve cardiovascular function in cardiac patients and the elderly.^33,34^ Considering that the 6-minute walk test simulates an over-ground walk, the current results support its use as an exercise mode to increase both daily physical activity and cardiorespiratory fitness in men and in patients with low cardiorespiratory fitness. However, in women and in patients with higher cardiorespiratory fitness, over-ground walking may not be enough to improve activity and fitness levels.

The cross-sectional design of this study is a limitation, as no causality can be inferred. Patients with severe cardiac disease and asymptomatic PAD or PAD more severe than claudication were excluded in the screening; therefore, the results can be extended only to our current sample of patients with claudication. Given we were not able to precisely identify VT in patients who stopped during the 6-minute walk test, generalization is also restricted to these patients. In addition, to accurately detect the VT in the 6-minute walk test, we only included patients that did not stop while performing it. These findings are also limited by the relatively small sample size, particularly when it comes to patients who did not achieve the VT.

## Conclusion

More than half of patients with symptomatic PAD achieved VT during the 6-minute walk test. Men and patients with lower cardiorespiratory fitness are more likely to achieve VT during the 6-minute walk test.
